# Operative management and outcomes in patients with myxomas: A single-center experience

**DOI:** 10.3389/fsurg.2023.1084447

**Published:** 2023-04-19

**Authors:** Ahmed Abdulfattah Alhasso, Okba F. Ahmed, Dana H. Mohammed-Saeed, Fahmi H. Kakamad, Saif S. Almodhaffer, Zaid A. Zaid, Hiwa O. Abdullah, Razhan K. Ali, Suhaib H. Kakamad, Diyar A. Omar, Berun A. Abdalla, Shvan H. Mohammed, Mohammed Q. Mustafa

**Affiliations:** ^1^ Department of Cardiac Surgery, Ibn Albittar Cardiac Surgeon, Baghdad, Iraq; ^2^ Mosul Cardiac Center, Mousl, Iraq; ^3^ College of Medicine, University of Sulaimani, Sulaimani, Iraq; ^4^ Scientific Affairs, Smart Health Tower, Sulaimani, Iraq; ^5^ Sulaimani Centre for Heart Disease, Sulaimani, Iraq; ^6^ Kscien Organization, Sulaimani, Iraq; ^7^ Surgery Department, Shar Hospital, Sulaimani, Iraq; ^8^ Medical Laboratory Technology, Shaqlawa Technical College, Erbil Polytechnic University, Erbil, Iraq; ^9^ Department of Medical Analysis, Tishk International University, Erbil, Iraq

**Keywords:** cardiac myxoma, cardiac tumor, cardiac surgery, case series, myxomas

## Abstract

**Background:**

Cardiac myxoma is a rare cardiac tumor that may be asymptomatic or can cause embolization or intracardiac obstruction, leading to heart failure, sudden cardiac death, and arrhythmia. This study aims to report an 11-year experience of a single center in the management of cardiac myxoma.

**Method:**

This study is a single-center retrospective case series. Eighty cases of cardiac myxoma were collected in Ibn Albitar's specialized center for cardiac surgery. Transthoracic echocardiography was used to make the preoperative diagnosis in all patients. The surgeries were undertaken through the standard approach of a median sternotomy. All four cardiac chambers were thoroughly explored for additional myxomas. The major objective of the operations was complete tumor resection.

**Result:**

The mean age of the patients was 46.3 years. Females (67.5%) were predominant over males (32.5%). Shortness of breath was the most common symptom (86.25%). The left atrium was the most affected site (83.75%), followed by the right atrium (13.75%). Coronary artery bypass grafting was required as the secondary or associated intervention in 19 (23.75%) cases. The recurrence rate was 11.25%, with a mortality rate of 3.75%.

**Conclusion:**

Recurrence and tumor embolism are risks of surgical intervention for myxoma. Good preparation using transthoracic echocardiography as a diagnostic tool and standard median sternotomy to complete resection of the tumors can decrease the rate of recurrence, embolism, and even mortality.

## Introduction

Primary cardiac tumors (PCT) are rare neoplasms that represent less than 0.2% of all tumors (1). Myxoma is a type of PCT that comprises 5%–10% of the cardiac and pericardial lesions. It most commonly affects females between the ages of 40 and 60 (2, 3). Generally, myxoma develops in the left atrium (LA), followed by the right atrium (RA) (4, 5). This tumor usually develops from the atrial septum near the fossa ovalis, but it can rarely develop from the posterior wall, anterior wall, or left atrial appendage (6). Based on the location, this neoplasm may be asymptomatic or can cause embolization or intracardiac obstruction, which results in heart failure, sudden cardiac death, and arrhythmia (4). However, surgical intervention has become the standard modality to manage cardiac myxoma, but intraoperative embolization and recurrence are the major complications (7). Therefore, surgical intervention is challenging and needs extensive care and experience to decrease morbidity and mortality (8, 9). This study aims to report an 11-year experience of a single center in the management of cardiac myxoma.

## Method

### Study design

This study is a single-center retrospective case series.

### Setting

Eighty cases of cardiac myxoma were collected in Ibn Albitar's specialized center for cardiac surgery for 11 years, from 2010 to 2021. The Iraqi board of medical specialization granted the study ethical approval.

### Inclusion criteria

This study enrolled those patients who underwent surgical excision of primary or recurrent intracardiac myxomas from 2010 to 2021.

### Exclusion criteria

Patients with acute thromboembolic ischemia, acute renal failure (on dialysis), or who were over 80 years old were excluded from this study.

### Data collection and analysis

All the required information on patients, including demographics, examination, medical summary, surgical indications, comorbidities, and follow-up, were recorded using an electronic registry database. The Statistical Package for the Social Sciences (SPSS) Version 25 was used to encode and perform descriptive analysis.

### Surgical intervention

The preoperative diagnosis was performed in all patients by transthoracic echocardiography (TTE). Coronary angiography was performed on patients over the age of 40. The operations were undertaken soon after the diagnosis of cardiac myxoma, through the standard surgical approach of a median sternotomy.

Cardiopulmonary bypass (CPB) with aorto-bicaval cannulation and mild hypothermia was done. Myocardial protection was achieved by cold St. Thomas and Del-Nido cardioplegia. The heart was not manipulated until the aorta had been cross-clamped to avoid systemic embolization. The surgical technique for LA myxomas was the right atrial transseptal or biatrial approaches. In order to decrease conduction problems, most of our operations were through the right atrium approach, so direct superior vena cava cannulation was an important technique, and the crisscross line suture connecting the right atrium to the inferior vena cava prevented the re-entry circuit.

The objectives of the operations were complete tumor resection with full-thickness removal of the tumor's attachment base and a cuff of the interatrial septum to prevent a recurrence. All four cardiac chambers were thoroughly explored for additional myxomas. The surgically defective sites were repaired with a Dacron patch because it is simple to handle and trim. Copious irrigation of the atria and ventricles with cold saline was done to eliminate any tumor fragments that might have been dislodged during the removal of the tumor. All the resected myxomas were subjected to routine histopathological examination. All the patients were followed up on an outpatient basis at regular intervals (3–6 months), and they underwent clinical examination, chest x-ray, electrocardiography, and echocardiography. To decrease the risk of atrial arrhythmias, a beta blocker was routinely used, and sometimes Cordarone was used pre- and post-operatively.

## Result

This study included 80 cases with either primary or recurrent intracardiac myxomas. The mean age of the patients was 46.3 years. Females (67.5%) were predominant over males (32.5%). Shortness of breath was the most common symptom (86.25%). The LA was the most affected site (83.75%), followed by the RA (13.75%) (Table 1). Coronary artery bypass grafting (CABG) was required as the secondary or associated intervention in 19 (23.75%) cases (Table 2). Atrial fibrillation (AF) was found in 6.25% of the cases. The mean time of intensive care unit (ICU) admission and hospital stays were 55 h and 9 days, respectively. Re-exploration (once) was done in 3.75% of the cases caused by bleeding. The recurrence and mortality rates were 11.25% and 3.75%, respectively (Table 3). Out of the recurrent cases, four (44.4%) were familial myxomas. The mortality in our cases was 30 days. One of them died after a concomitant procedure (mitral valve replacement) due to low cardiac output (right ventricular failure), and another one died after primary surgery caused by renal impairment. The third one died after reoperation due to a cerebral vascular accident (CVA). During the average 5 years of follow-up, an echocardiogram was done every three months for patients with a positive family history and every six months for patients with a negative family history.

**Table 1:** The baseline characteristics of the patients with myxoma.

Baseline characteristics	Number/percentage
Mean age (years)	46.3 ± 1.277, CI*: 95%
**Sex**	
Male	26 (32.5%)
Female	54 (67.5%)
**Medical conditions**	
Diabetes mellitus	23 (28.75%)
Shortness of breath	69 (86.25%)
Atrial fibrillation	5 (6.25%)
Palpitations	7 (8.75%)
Hypertension	33 (41.25%)
Familial myxoma	7 (8.75%)
Cerebrovascular accident	5 (6.25%)
Severe mitral regurgitation	6 (7.5%)
**Tumor location**	
Left atrium	67 (83.75%)
Right atrium	11 (13.75%)
Both atrium	1 (1.25%)
Right ventricle	1 (1.25%)

CI, confidence level.

**Table 2:** Intraoperative data.

Variables	Number/percentage
Mean time of aortic cross-clamping (minutes)	46.14 ± 1.31, CI*: 95%
Mean time of Cardiopulmonary bypass (minutes)	90.74 ± 1.37, CI*: 95%
**Cardioplegia**	
St. Thomas	70 cases (87.5%)
Del-Nido	10 cases (12.5%)
**Concomitant procedure**	
Coronary artery bypass graft (CABG)	19 (23.75%)
Mitral Valve Regurgitation (MVR) replacement	14 (17.5%)
Tricuspid regurgitation (TR) repair	5 (6.25%)

**Table 3:** Post-operative and follow-up outcomes.

Variables	Numbers/percentage
**Post-operative outcomes**	
ICU stay (mean)	55 h ±1.552, CI*: 95%
Hospital stay (mean)	9 days ±0.355, CI*: 95%
Mortality	3 (3.75%)
Wound infection	4 (5%)
Re-exploration	3 (3.75%)
Atrial fibrillation (AF)	5 (6.25%)
Renal impairment	3 (3.75%)
**Follow-up outcomes**	
Recurrence	9 (11.25%)

CI, confidence level.

## Discussion

Myxoma is the most common type of benign cardiac tumor that originates from primitive stromal cells and differentiates along the endothelial lines (3, 10). This tumor usually develops in the LA; however, its occurrence in other sites like the RA, left ventricle, mitral, and tricuspid valves has been reported (10, 11). Females between the ages of 40 and 60 are more frequently affected by myxoma, except for familial myxoma, which is commonly seen in young-aged individuals (3, 10). In the current series, females were the dominant group, and the mean age of the patients was 46.3 years. The LA was found to be the affected site in 83.75% of the cases, followed by the RA (13.75%). There was only one case of biatrial myxoma and one of right ventricular myxoma.

Alongside the hemodynamic abnormalities owing to the embolism or tumor obstruction, several constitutional symptoms may be observed in patients with myxoma, including dyspnea, palpitation, fever, and weight loss. The clinical manifestations of the tumor are determined by its size, location, surface, and mobility (1, 3). Dyspnea (70%) and palpitation (35%) have been reported to be the most familiar symptoms (4, 12). An embolism caused by myxoma may result in myocardial infarction (13). Therefore, symptomatic myxomas should be surgically excised immediately after diagnosis to prevent pulmonary embolism and other complications (1). The incidence of pulmonary embolism and systemic embolization from left atrial myxomas has been reported to be less than 10% and 25%–50%, respectively. Another study reported cerebrovascular accidents (CVA) in 22% of the cases (3, 14). In this study, most of the cases (86.25%) presented with shortness of breath (dyspnea), but a small number of patients (8.8%) had palpitations. CVA was found to be 6.3%, and none of the cases were associated with systemic or pulmonary embolism.

Different techniques have been proposed to diagnose cardiac myxoma. Cardiac catheterization and angiocardiography were originally used to diagnose atrial myxoma, but this traditional method is an invasive procedure that can cause peripheral embolism and may provide false-positive or false-negative results (7). Echocardiography is a noninvasive and accurate procedure to detect intracardiac tumors. This modality can determine the character, location, and mobility of cardiac myxoma, with the capability to consider the presence of biatrial tumors and multiple carcinomas. TTE has a sensitivity of 95% in detecting myxoma (4, 7). In open-heart surgical interventions, ischemic cardiac arrest by cardioplegic solutions and cardiac hypothermia have been encouraged, especially in the excision of atrial myxoma. This application provides a relaxed field for operation and decreases the risk of tumor embolism (7). The recent surgical method in the treatment of atrial myxomas is the standard median sternotomy with CPB, mild hypothermia, and cardioplegic solutions (2, 15). In the present study, TTE was conducted in all patients preoperatively, and coronary angiography was also performed in patients older than 40 years. CPB and mild hypothermia were used, and myocardial protection was achieved by cold St. Thomas and Del-Nido cardioplegia solutions. All surgical operations were conducted through the standard median sternotomy.

Selkane et al. in their case series reported six concomitant mitral valve procedures with three cases (7.5%) of secondary replacement of the mitral valve (16). Furthermore, Kabbani et al. conducted four (16.6%) mitral valve replacements in the resection of 24 myxoma tumors. In a study by Semb et al., tricuspid valve replacement was performed in 18.18% of the cases (17, 18). In this study, CABG was performed in 23.75% of the cases, and mitral valve replacement and tricuspid valve repair were conducted in 17.5% and 6.25% of the cases, respectively.

To reduce the risk of recurrence, the myxoma must be completely removed by resection of the tumor's base and a portion of the surrounding interatrial septum (1). The recurrence rate of atrial myxoma has been reported to be 5% to over 14% (6). Several factors, like family history of myxoma, young age, multifocal tumors, and interleukin-6, play a significant role in the recurrence of myxoma (6, 16). Postoperatively, the recurrence in patients with familial myxoma is 21%–67% (19, 20). In previous studies, the mortality rate due to the excision of myxoma has been quite variable. In a study by Sutton et al., the mortality rate was 2.7%, and 5% mortality has also been reported (2, 21). A high mortality rate of about 25% was reported in a series of 17 cases (22). Symbas et al. recorded a high mortality rate in their study due to embolization and obstruction of intracardiac blood flow in the time interval between diagnosis and operation (23). In the current study, total resection of the tumors was conducted. Irrigation of the atria and ventricles with cold saline was done to eliminate any tumor fragments and decrease the risk of recurrence. However, the recurrence rate in this series was 11.25%, and nearly half of them were familial myxomas; this may be an explanation for this rate of recurrence in this study. The mortality rate was 3.75%, which is a fair outcome in comparison to the previously mentioned studies.

## Conclusion

Recurrence and tumor embolism are major risks of surgical intervention for myxoma. Good preparation using TTE as a diagnostic tool and conducting a standard median sternotomy by experienced surgeons to complete resection of the tumors can decrease the rate of recurrence, embolism, and even mortality rate.

## Data Availability

The original contributions presented in the study are included in the article/supplementary material, further inquiries can be directed to the corresponding author/s.
